# Case report: Trigeminal neuralgia misdiagnosed as glossopharyngeal neuralgia

**DOI:** 10.3389/fneur.2023.1079914

**Published:** 2023-01-19

**Authors:** Liangzhe Wu, Jinbiao Xiong, Ying Huang, Kunning Han, Kunhao Cai, Xuejun Fu

**Affiliations:** ^1^The Second Clinical Medical College, Jinan University, Shenzhen, Guangdong, China; ^2^Department of Neurosurgery, Shenzhen People's Hospital (The Second Clinical Medical College, Jinan University, The First Affiliated Hospital, Southern University of Science and Technology), Shenzhen, Guangdong, China; ^3^Department of Neurology, Shenzhen People's Hospital (The Second Clinical Medical College, Jinan University, The First Affiliated Hospital, Southern University of Science and Technology), Shenzhen, Guangdong, China

**Keywords:** trigeminal neuralgia, glossopharyngeal neuralgia, microvascular decompression, magnetic resonance imaging, misdiagnosed

## Abstract

**Background:**

Trigeminal neuralgia (TN) and glossopharyngeal neuralgia (GPN) are cranial nerve neuralgias with the same clinical manifestations, pathological features, and trigger factors; their affected sites are adjacent. Performing a magnetic resonance imaging (MRI) examination alone can easily lead to a misdiagnosis.

**Case presentation:**

A 72-year-old man had visited another hospital with severe left-sided tongue pain. On MRI, vascular compression of the glossopharyngeal nerve had been visible, with unclear evidence of trigeminal nerve involvement. He had been diagnosed with left-sided GPN and underwent microvascular decompression (MVD) of the left glossopharyngeal nerve. However, no improvement was observed after surgery. During a second surgery at our hospital, MVD of the trigeminal nerve was performed, and the trigeminal nerve was fully explored and separated. The patient's pain resolved after surgery. Ultimately, the patient was definitively diagnosed with left-sided TN.

**Discussion and conclusion:**

MVD is currently the most efficacious surgical option for treating cranial nerve neuralgia. To select patients for MVD, having an MRI criteria for identifying true neurovascular compression will be helpful. However, clinicians should focus more on a patient's clinical symptoms and not rely solely on MRI findings. This patient's case can help clinicians distinguish between TN and GPN, improve the understanding of these diseases, avoid misdiagnosis, and reduce the possibility of secondary damage.

## 1. Introduction

Trigeminal neuralgia (TN) is a chronic neuropathic pain disorder that occurs mainly in the face, especially in the cheeks and corners of the mouth, and rarely in the tongue, mouth, and jaw. Glossopharyngeal neuralgia (GPN) is a rare facial syndrome characterized by pain in the throat, tonsils, and tongue, with occasional pain in the mandibular angle and ear. Both TN and GPN can occur spontaneously or can have specific precipitating factors. The most common etiology involves demyelinating lesions induced by vascular compression of nerves in the brainstem; this is known as the root entry zone (REZ). The antiepileptic drugs carbamazepine and oxcarbazepine are the first-line treatment options; if drug therapy is ineffective or the patient cannot tolerate the side effects, microvascular decompression (MVD) is the preferred surgical option. When a patient presents with tongue pain and magnetic resonance imaging (MRI) shows intracranial vascular compression of the glossopharyngeal nerve, it is easy to consider a diagnosis of GPN but not TN. A misdiagnosis potentially results in multiple surgeries; therefore, the ability to distinguish between the two diseases is essential. To the best of our knowledge, the present case is the first case of TN misdiagnosed as a GPN.

## 2. Case description

**Figure 1:**
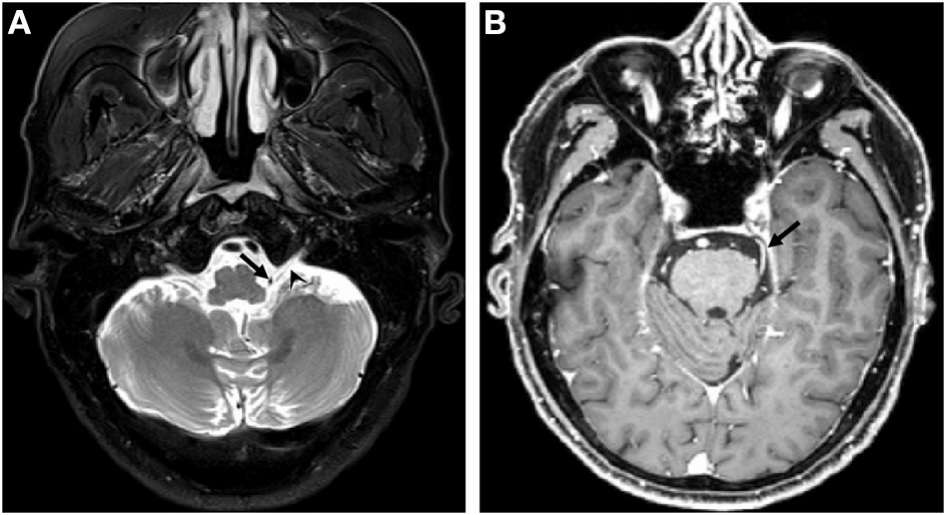
MRI before the first operation. **(A)** The posterior inferior cerebellar artery (arrow) touches the left glossopharyngeal nerve (arrowhead); **(B)** The SCA (arrow) compression of the left trigeminal nerve is not displayed clearly. SCA, superior cerebellar artery.

A 72-year-old man was admitted to the hospital with intermittent severe pain in the left middle one-third of the tongue for 8 months, triggered by tongue movements and chewing of food, lasting from a few to dozens of minutes at a time, and was treated effectively with carbamazepine. Swallowing did not cause any pain. There was no pain at the face, base of the tongue, throat, or tonsils, and no other neurological symptoms. Four months prior, the patient underwent an MRI exam (Figure 1) and was diagnosed with left-sided GPN; he was treated with glossopharyngeal nerve MVD at another hospital. The patient's symptoms did not improve after surgery. The day after the surgery, the pain recurred to the preoperative condition and gradually aggravated. On the third post-operative day, there was clear fluid outflow from the nose, suggesting cerebrospinal fluid rhinorrhea. In addition, the patient developed reduced hearing in the left ear. The symptoms of the clear fluid outflow from the nose disappeared 10 days after the surgery.

**Figure 2:**
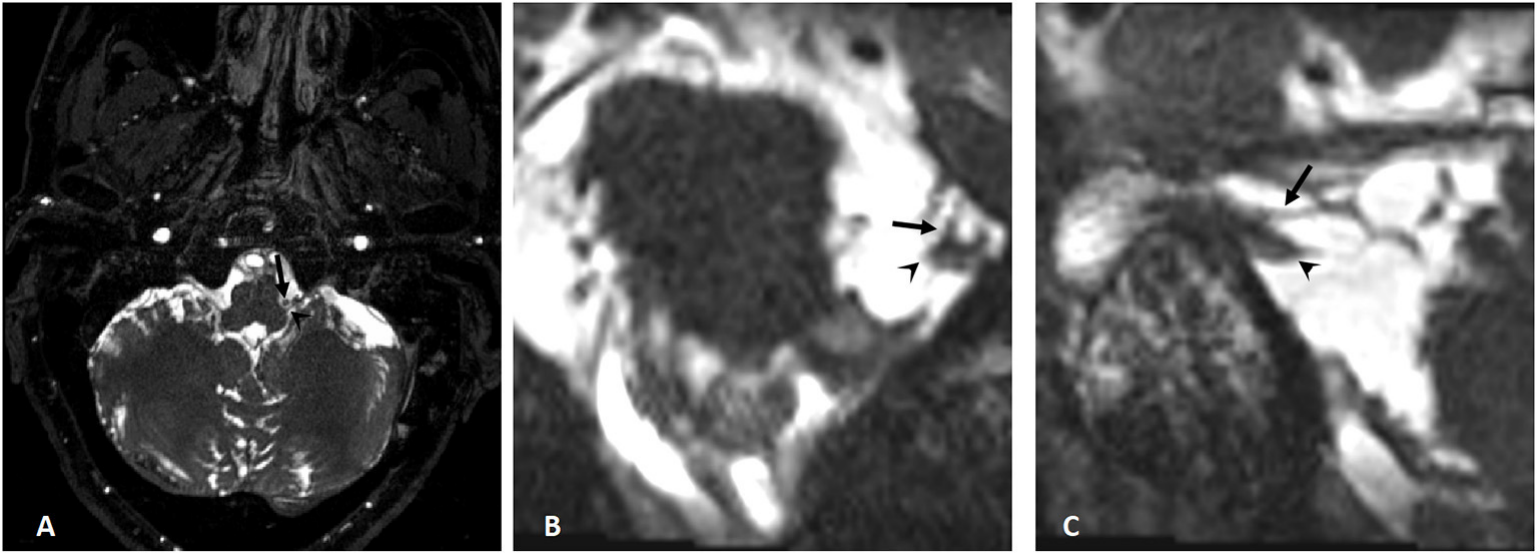
MRI before the second operation. **(A)** The structure around the left glossopharyngeal nerve is disordered, and the original posterior inferior cerebellar artery (arrow) seems to be moved with no compression to the left glossopharyngeal nerve (arrowhead); **(B, C)** The left trigeminal nerve (arrowhead) touches the superior cerebellar artery (arrow).

**Figure 3:**
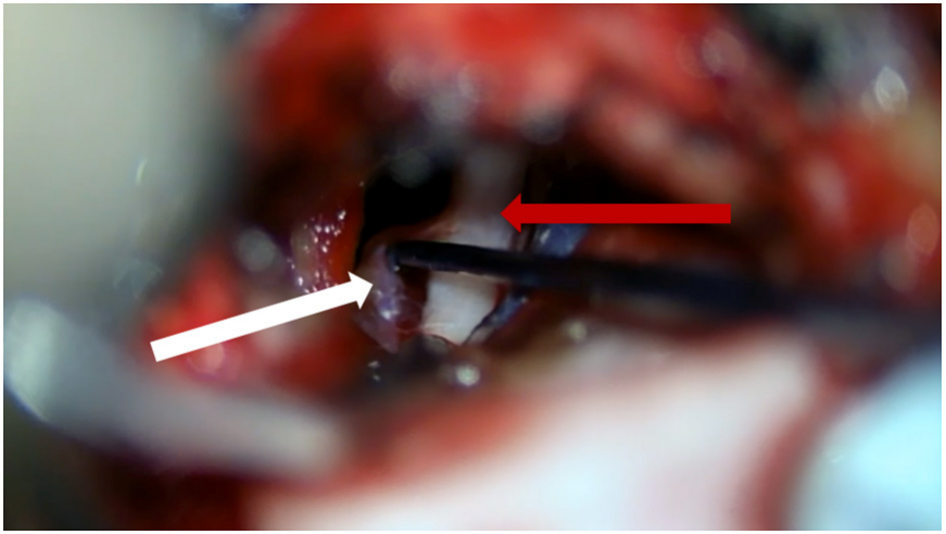
Teflon sponges (white arrow) separate small blood vessels away from the trigeminal nerve (red arrow).

**Figure 4:**
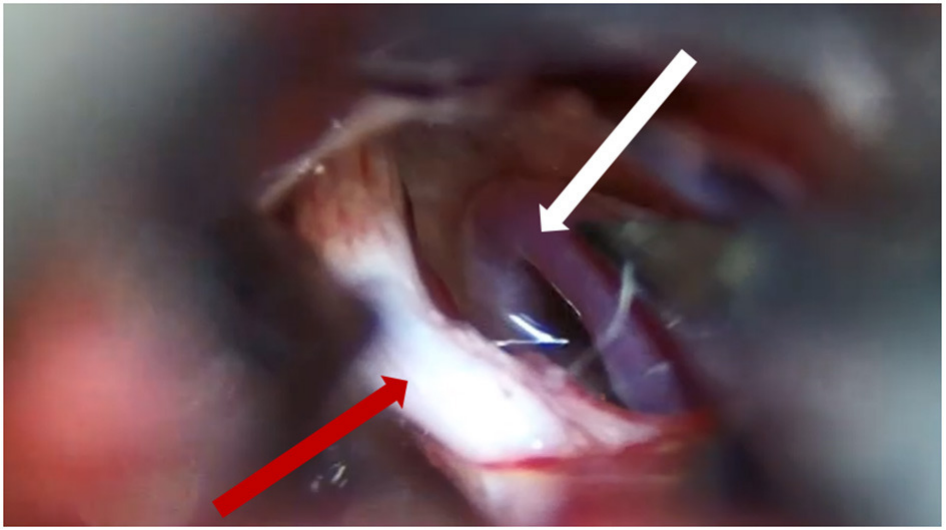
The SCA (white arrow) compresses the trigeminal nerve (red arrow). SCA, superior cerebellar artery.

Following admission to our hospital, the patient had a second MRI exam (Figure 2). The patient underwent a second surgery for cerebellopontine angle exploration (CPA). The previous craniotomy was reopened in the lateral position, and the dura was cut open. While exploring the trigeminal nerve area, we observed a small blood vessel close to the REZ with a Teflon sponge adhering to it (placed during the first operation) (Figure 3). The vessel was displaced without compressing the trigeminal nerve. The blood vessel was small; therefore, it was not considered to be the responsible vessel. The superior pole of the trigeminal nerve was then examined. After cutting arachnoid adhesions, the superior cerebellar artery (SCA) was found to be significantly suppressing the REZ area of the trigeminal nerve. Following the placement of a Teflon sponge between the vessel and nerve to make a total separation, we continued to explore the trigeminal nerve near Meckel's cave. We saw that the SCA was turned forward and downward, oppressing the trigeminal nerve (Figure 4), which was separated completely by the Teflon sponge. Finally, the space between the glossopharyngeal nerve and the facial acoustic nerve was explored; the glossopharyngeal nerve anatomy was unclear, and there was no apparent vascular compression. Therefore, no further dissections were performed. The dura mater was sutured in a watertight pattern, and the wound was closed in layers. Postoperatively, the patient's pain resolved completely. No new neurological deficits or surgery-related complications were observed.

## 3. Diagnostic assessment

In the sensory innervation of the tongue, the mandibular branch of the trigeminal nerve innervates the anterior two-thirds of the tongue, and the glossopharyngeal nerve innervates the posterior one-third of the tongue. The pain in the middle one-third of this patient's tongue was located in the demarcation zone of the trigeminal and glossopharyngeal nerves. Anatomically, this area is involved in the innervation of the trigeminal nerve, but symptoms did not improve after the first surgery. When drug therapy is ineffective, MVD is currently the best surgical plan to treat cranial nerve neuralgia caused by intracranial vascular compression of nerves. Studies report that the success rate of MVD is 83.4% for TN and 97.4% for GPN (1, 2). When postoperative ineffectiveness occurs, we must consider that certain areas were missed during exploration or the diagnosis may be incorrect. Although the trigeminal nerve was not displayed clearly on the patient's MRI, the MRI's negative-predictive value was only 33.3%; thus, TN was still considered. We fully explored and separated the responsible vessels of the trigeminal nerve during surgery, and the tongue pain disappeared after surgery. The trigeminal nerve is a large sensory rootlet that exits the lateral aspect of the midpons medial to the middle cerebellar peduncle; the ophthalmic division is the most inferior, the maxillary division is in the middle, and the mandibular division is in the superior position (3). The trigeminal nerve was compressed from above to below by the SCA during surgery and was therefore diagnosed more definitively as left-sided TN.

## 4. Discussion

The patient had simple pain in the left middle third of the tongue, without pain in the root of the tongue or throat, and without swallowing dysfunction or other neurological abnormalities. In addition, he had previously undergone MVD of the left GPN.

The etiology of TN can be divided into three categories: classic, secondary, and idiopathic. The classical type is intracranial vascular compression upon the REZ of the trigeminal nerve. Secondary TN, combined with other neurological symptoms, is commonly associated with multiple sclerosis and tumors in the pontocerebellar region. Idiopathic TN indicates that no neurological cause can be found (4). The incidence of GPN is approximately 1/100th that of TN (5). GPN is divided into idiopathic and secondary types. Idiopathic neuralgia has no clear etiology. Secondary causes include intracranial vascular compression, tumors, multiple sclerosis, trauma, and Eagle's syndrome. The differential diagnosis of TN and GPN depends mainly on different clinical manifestations, with MRI performed as an auxiliary diagnosis. The pain in TN occurs in the area controlled by the trigeminal nerve, including the face, tongue, and jaw; trigger points are also present in this area. In GPN, the pain ranges among the auricular and pharyngeal branches of the glossopharyngeal and vagus cranial nerves, including the ear, pharynx, root of the tongue, and mandibular angle; the pain can be spontaneous or induced by swallowing, chewing, coughing, talking, and yawning (6). A cranial MRI exam is the first choice for evaluating the contact between blood vessels and nerves. Preoperative MRI sensitivity is 75.8%, with a specificity of 65.8%, and positive- and negative-predictive values of 92.4 and 33.3%, respectively, for MVD (7).

GPN is often misdiagnosed as TN, nervus intermedius neuralgia, myofascial pain dysfunction syndrome, pharyngitis, or tonsillitis. The main reasons for misdiagnosing the glossopharyngeal nerve as TN include that (1) GPN is much rarer and less recognized than TN, accounting for 1/100th of TN cases; (2) diagnosis of cranial nerve neuralgia is dependent mainly on symptoms and signs; (3) when the patient history description is not accurate, a lack of a specific diagnostic basis may exist; and (4) being misled is easy. For example, some patients with GPN subjectively describe experiencing pain when chewing and eating. However, this symptom does not differentiate GPN from TN. In addition, if clinical experience is lacking when the medical history is unknown, a misdiagnosis can easily occur.

Having many branches, the glossopharyngeal nerve is widely distributed. The glossopharyngeal nerve, vagus nerve, accessory nerve, and hypoglossal nerve all originate from the medulla oblongata, with some branches of the glossopharyngeal nerve also entering the spinal nucleus of the trigeminal nerve. Therefore, the pain range may extend to the distribution area of the trigeminal nerve or be complicated by TN. When the glossopharyngeal nerve is damaged, the symptoms of damage to the vagus and hypoglossal nerve can coincide; the location of the pain can also change. Therefore, making a diagnosis is difficult. This phenomenon occurs in nearly 20% of GPN cases. MRI has a high predictive value for vascular–nerve contact, but its false-positive and false-negative rates may lead to a misdiagnosis or missed diagnosis.

Interestingly, the opposite occurred in this case: TN was misdiagnosed as GPN. The main cause of the misdiagnosis was that the patient showed simple pain in the middle one-third of the left tongue without other concomitant symptoms. The pain was located in the adjacent innervation areas of the trigeminal and glossopharyngeal nerves; this does not occur in the classically described TN and GPN. The cranial MRI exam indicated that the glossopharyngeal nerve was compressed, but the trigeminal nerve was not displayed clearly. These MRI results, combined with the patient's medical history, facilitated a misdiagnosis of GPN; the positive-predictive rate of MRI is 92.4%, which is not optimal for diagnosis. Because cranial nerve neuralgia is diagnosed mainly by symptoms and signs, inadequacies in the medical history and physical examination can easily lead to misdiagnosis. If the preoperative diagnosis is unclear, and the vessels responsible for the suspected nerve damage have not been determined, the nerves should be fully explored and separated.

TN and GPN are the types of cranial nerve neuralgia with the same clinical and pathological features and trigger factors, with the pain locations overlapping the two groups of nerves. Because of this, misdiagnosis often occurs. Therefore, it is essential to identify differences between TN and GPN for proper diagnosis and treatment.

In this case, although the trigeminal nerve was separated in the first surgery, it was not carefully explored and separated, so the main responsible vessels were not identified. As a result, the pain persisted after the first surgery. In the second surgery, the trigeminal nerve was explored and decompressed fully, and the postoperative outcomes were good. Therefore, it is necessary to fully explore the trigeminal nerve and separate all potentially responsible vessels during surgery. Although preoperative MRI has a high positive-predictive value and sensitivity, a relatively low negative-predictive value reduces its usefulness in patients without vascular compression of nerves from MVD. Therefore, performing MVD should be coordinated with clinical manifestations and imaging; many MRI patients with negative compression may still benefit from MVD (7).

The diagnosis of cranial nerve disease is based primarily on clinical manifestations. Therefore, a patient's medical history should be reviewed thoroughly before surgery, and the target nerve should be determined. MRI should be performed only as a clinical diagnostic support and not as a determinant. If it is difficult to determine where the cranial nerve pain originates, a full exploration and complete separation of suspicious nerves should be performed simultaneously.

## 5. Conclusion

Misdiagnosis can easily occur in the area adjacent to the innervation areas of the trigeminal and glossopharyngeal nerves. For neuralgia with vascular compression, the patient's medical history should be investigated carefully, the target nerve should be determined, and full exploration and separation of the target nerves should be performed to avoid the risk of failure and recurrence. Although MRI is the primary method used to identify neurovascular compression, providing operators with a preoperative evaluation and choice of operation methods, it is only an auxiliary means. Therefore, a patient's clinical symptoms should be identified first, and basic clinical and physical examinations should not be ignored. When a diagnosis is highly suspicious and the MRI exam yields unclear results, MVD should be performed using full exploration.

## Data availability statement

The original contributions presented in the study are included in the article/Supplementary material, further inquiries can be directed to the corresponding authors.

## Ethics statement

Written informed consent was obtained from the individual(s) for the publication of any potentially identifiable images or data included in this article.

## Author contributions

LW wrote the manuscript and was involved in the diagnostic and therapeutic clinical progress. JX was involved in the patient's care. KH supported the case interpretation. KC revised the manuscript for intellectual content and was responsible for patient diagnosis and treatment. XF contributed to the figures, helped with the diagnostic process, and critically revised the manuscript. All authors contributed to the manuscript and approved the submitted version.
